# Phase change nanodroplets: Challenges and clinical potential

**DOI:** 10.1016/j.apsb.2026.05.030

**Published:** 2026-05-28

**Authors:** Xiaoyu Wang, Roman Barmin, Roger Molto Pallares, Anne Rix, Fabian Kiessling

**Affiliations:** Institute for Experimental Molecular Imaging (ExMI), RWTH Aachen University, Aachen 52074, Germany

**Keywords:** Nanodroplets, Phase change, Ultrasound, Biological behavior, Molecular imaging, Drug delivery, Theranostics

## Abstract

Phase change nanodroplets (PCND) consist of a liquid fluorocarbon core, which vaporizes into gas upon energy excitation, and a stabilizing shell. Through this phase-change property, PCND combine nanoparticle-like stability and pharmacokinetics, such as tissue extravasation and uptake by the mononuclear phagocyte system, with distinctive microbubble-like capabilities, namely ultrasound contrast enhancement and energy-triggered therapeutic action. PCND serve both as highly sensitive imaging agents, from molecular imaging to high-resolution vascular mapping, and as versatile therapeutics, used for example for thrombolysis, tissue ablation, biofilm removal, immunomodulation, and drug delivery. This review article provides a focused review of the biological behavior of PCND, including blood half-life, biodistribution, clearance pathways, and tumor accumulation, following a brief overview of the key physicochemical features that shape their *in vivo* fate. By comparing data across studies, we identify major inconsistencies in reported pharmacokinetics and highlight critical knowledge gaps, particularly regarding safety data. We further summarize advances in PCND-enabled imaging and therapy, and propose strategies to strengthen formulation design, biological evaluation, and stimulation protocols to support future clinical translation. Overall, PCND hold strong potential as a versatile theranostic platform. Continued systematic and clinically focused research is expected to accelerate their path toward clinical translation.

## Graphical abstract

This review analyzes formulation-dependent pharmacokinetics and safety of phase change nanodroplets, identifies key inconsistencies and knowledge gaps, and proposes design and evaluation strategies to accelerate clinical translation in advanced applications.

**Figure:**
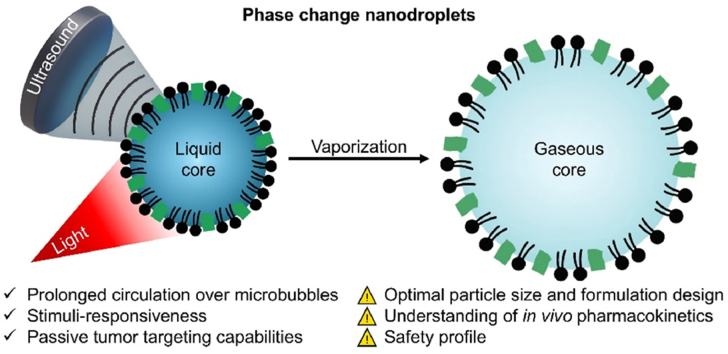

## 1 Introduction

Ultrasound is used for many different purposes, ranging from synthetic chemistry to clinical imaging and therapy^1^. In medical ultrasound, the introduction of microbubbles has significantly improved diagnostic accuracy by providing information on microvascular blood flow in organs and diseased regions^2^. In addition, the oscillation of microbubbles during ultrasound exposure can induce cavitation, shear forces, and microstreaming, promoting transient opening of biological barriers such as tumor endothelium or the blood–brain barrier through a process known as sonopermeation, thereby enhancing drug accumulation in the targeted tissue. However, their relatively large size (1–10 μm), which prevents their extravasation into tissues and limits their circulation times to several minutes, has prompted interest in smaller nanoparticles as an alternative^3^. Nanoparticles, with sizes typically ranging from 5 to 200 nm, can circulate for longer times (hours to days) and accumulate in tumor tissues through mechanisms such as the enhanced permeation and retention (EPR) and the active transport and retention (ATR) effects^4^. However, most nanoparticles do not exhibit significant acoustic response, limiting their potential for ultrasound imaging and therapy^5^.

Therefore, phase change nanodroplets (PCND) have been developed as a hybrid solution, combining the prolonged circulation and tissue penetration of nanoparticles with the acoustic properties of microbubbles. This topic has seen rapidly growing interest in recent years (78 publications in 2023, 119 in 2024, and 136 in 2025, as of middle December 2025). Structurally, PCND have diameters of 50–1000 nm and consist of a liquid fluorocarbon core coated with a stabilizing shell. Upon external excitation, the liquid core can vaporize and form a gaseous microbubble that is significantly larger (up to 5 times in diameter)^6^ than the original PCND.

In this review, we first outline the physicochemical properties of PCND, as an essential basis to understand their working principle and functional performance. Next, we discuss the impact of their formulation design on their biological behavior regarding blood half-life, organ biodistribution, and clearance pathways, followed by a comparison of PCND with microbubbles and conventional nanoparticles in these aspects. Finally, we discuss the current limitations of PCND that hinder clinical translation and suggest strategies to overcome them.

## 2 Chemical composition of PCND and functionalization toolbox

**Figure 1:**
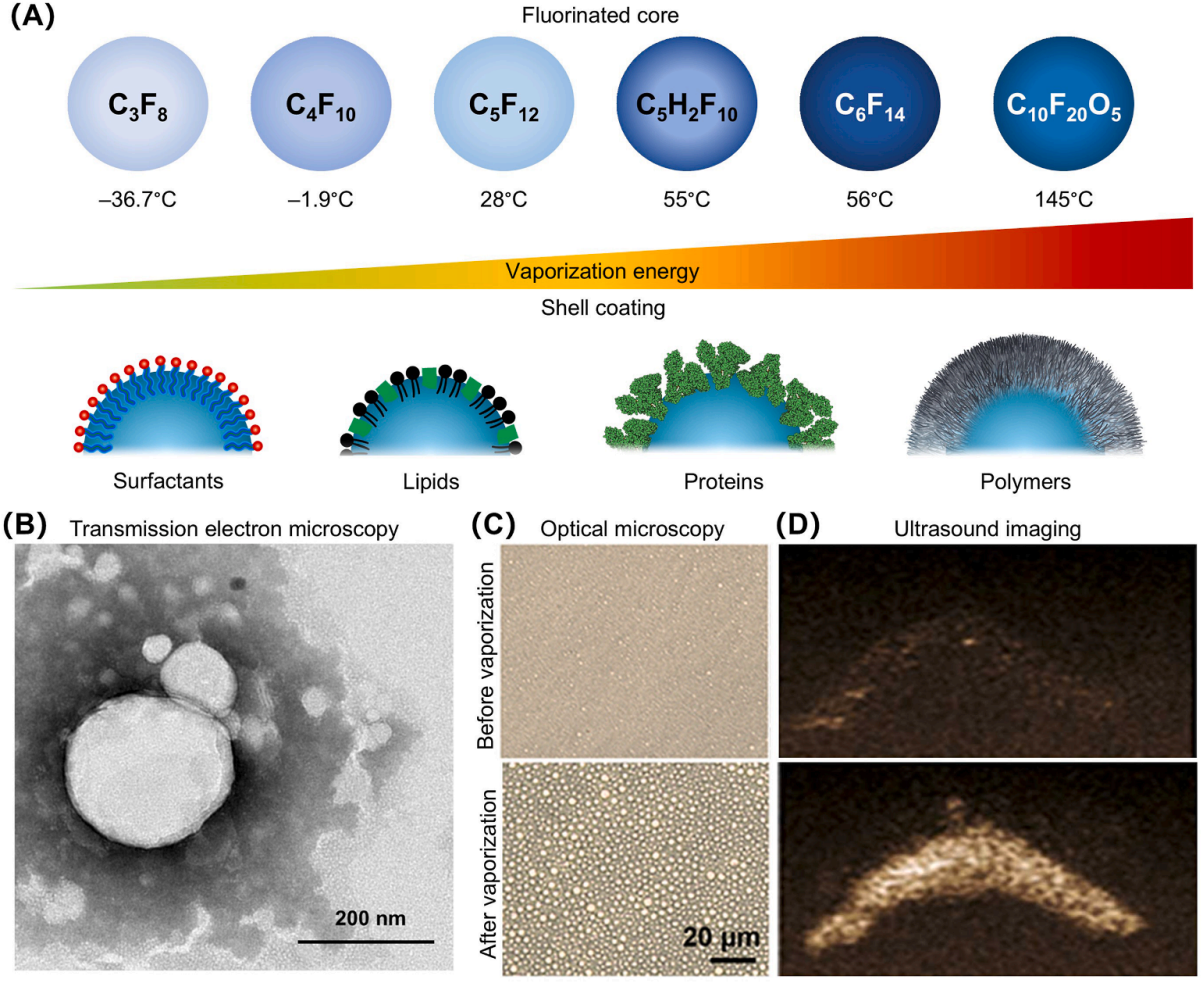
Chemical composition and characterization of phase-change nanodroplets (PCND). (A) Schematic illustration of core and shell materials used in PCND designs. Representative fluorinated core compounds are shown as spheres, labeled with their boiling points and color-coded from light to dark blue to indicate increasing vaporization energy required for phase transition. Each core is encapsulated within a shell composed of surfactants, lipids, proteins, or polymers, which can further modulate the vaporization energy depending on their composition and interfacial properties. (B) Transmission electron microscopy image of a C_6_F_14_-core PCND with a polymer shell. Reproduced from Ref. 17, under CC-BY-NC-ND license. (C) Phase contrast microscopy images of PCND before and after ultrasound exposure, showing phase size changes. Reproduced from Ref. 18, under CC BY-4.0. (D) Contrast-mode ultrasound images showing laser-induced vaporization of PCND and enhanced echogenicity in an *ex vivo* spleen model. Reproduced from Ref. 10, under CC-BY-NC-ND license.

The fluorocarbons used in PCND synthesis cover a wide boiling point range (from −36.7 to 145 °C) but remain in a metastable liquid state under storage conditions. This stability is reinforced by the shell, which imposes Laplace pressure around the liquid core^7^. Additionally, the homogeneous nucleation theory has been proposed to explain their exceptional stability, as the formation of a critical vapor nucleus requires overcoming a substantial free-energy barrier that prevents spontaneous vaporization^8^. Although PCND remain stable under ambient conditions, this metastability makes them highly responsive to external energy inputs. Under exposure to sufficient acoustic or radiation pressure (from ultrasound or laser pulses, respectively), these formulation characteristics ultimately determine the phase transition process known as acoustic or optical droplet vaporization (ADV or ODV)^9^, ^10^, ^11^. In ADV, the negative pressure phase of the ultrasound wave transiently reduces the total pressure acting on the PCND—consisting of ambient, Laplace, and acoustic components—below the vapor pressure of the fluorocarbon core, triggering a liquid-to-gas transition and generating mechanical forces^12^. In ODV, laser-induced heating of embedded absorbers raises the temperature of PCND above the vaporization threshold, producing microbubbles detectable by ultrasound^13^. Vaporized PCND primarily undergo sustained inertial cavitation^14^^,^^15^. This contrasts with microbubbles, where both stable and inertial cavitation mechanisms are involved in ultrasound imaging and therapy strategies^16^. Because the vaporization threshold of PCND depends on both external parameters (*e.g*., transducer frequency of ultrasound, wavelength of laser) and intrinsic formulation parameters (*e.g.,* boiling point of fluorocarbon core, shell composition), understanding the physicochemical properties of PCND is important. This section therefore reports core and shell materials involved in PCND formulations (illustrated in Fig. 1A) and discusses how these components influence the properties of PCND. In addition, we provide examples of a typical PCND characterization workflow, including a transmission electron microscopy micrograph of a PCND^17^, optical micrographs captured before and after vaporization^18^, and representative ultrasound images^10^ (shown in Fig. 1B–D, respectively). These examples highlight the potential for standardizing the PCND characterization pipeline.

### 2.1 Fluorinated core

The selection of the fluorinated core material is critical for determining the stability and phase transition behavior of PCND. Fluorocarbons are small molecules composed of strong carbon–fluorine (C–F) bonds and exhibit low overall polarity due to their symmetrical structure, which contributes to bioinertness^19^ and hydrophobicity^20^. While short-chain fluorocarbons exhibit weak intermolecular forces and low boiling points, increasing molecular weight leads to higher boiling points due to stronger van der Waals interactions^20^. The most commonly used fluorocarbons in PCND synthesis include perfluoropropane (C_3_F_8_), perfluorobutane (C_4_F_10_), perfluoropentane (C_5_F_12_), and perfluorohexane (C_6_F_14_), with boiling points ranging from −39 °C to 56 °C. Table 1 summarizes fluorocarbons, used as core material, and the resulting PCND formulations. Rational selection of the fluorocarbon core enables fine-tuning of vaporization thresholds and optimization of ultrasound responsiveness^14^^,^^21^. Fluorocarbon blends (*e.g.*, C_4_F_10_/C_5_F_12_^22^ or C_5_F_12_/C_5_H_2_F_10_^23^) allow modulation of vaporization thresholds and stability by altering intermolecular interactions and phase change dynamics, leading to improved control over ultrasound triggered activation. In addition to fluorocarbon, fluorinated ethers, such as C_10_F_20_O_5,_ have emerged as promising core materials for PCND, offering a balance of low surface tension and suitable volatility that supports stable droplet formation and efficient ultrasound-triggered phase transition^24^.

**Table 1:** Summary of PCND compositions based on the choice of fluorocarbon used for the core material, and the shell of different types.

Core material	Molecular weight	Boiling point (°C)	Shell composition	Mean diameter	Ref.
C_3_F_8_	188.2	−36.7	Lipid	100–200 nm	35,36
			Lipid	250–350 nm	37
C_4_F_10_	238.3	−1.9	Lipid	100–200 nm	38, 39, 40
			Lipid	200–300 nm	15,41,42
			Lipid	<1 μm	43
			Lipid + surfactant	300–400 nm	44
			Lipid	500–600 nm	45
			Polymer	700–800 nm	46
C_5_F_12_	288.03	28	Lipid	<100 nm	47
			Lipid	100–200 nm	48,49
			Lipid	200–300 nm	50
			Lipid	300–400 nm	51, 52, 53
			Lipid	400–500 nm	25,54
			Surfactant	200–300 nm	55
			Surfactant	300–400 nm	10
			Polymer	100–200 nm	56, 57, 58
			Polymer	200–300 nm	18
			Polymer	300–400 nm	34
			Polymer	700–800 nm	59,60
C_5_H_2_F_10_	252.05	55	Lipid	300–400 nm	54
			Polymer	Peak at 160,720 nm	61
C_6_F_14_	338.04	56	Lipid	100–200 nm	14
			Lipid	200–300 nm	62
			Lipid	300–400 nm	63
			Surfactant	100–200 nm	64
			Surfactant	300–400 nm	65,66
			Polymer	200–300 nm	17
			Polymer	<100 nm	60
			Hybrid (Lipid and polymer)	200–300 nm	67
C_10_F_20_O_5_	580.07	145	Polymer	100–200 nm	68
			Polymer	200–300 nm	24
			Polymer	300–400 nm	61

### 2.2 Shell materials

The shell material of PCND—typically composed of lipids, surfactants, proteins, or polymers—serves as a protective barrier that prevents rapid core dissolution and multiparticle coalescence (Fig. 1A).

Lipid-based shells, primarily made from phospholipids, such as 1,2-dipalmitoyl-*sn*-glycero-3–phosphocholine (DPPC) and 1,2-distearoyl-*sn*-glycero-3-phosphocholine (DSPC), offer excellent biocompatibility and shell elasticity^25^. These properties provide a favorable balance between stability and ultrasound responsiveness, making lipid shells well-suited for applications requiring efficient ultrasound-triggered vaporization.

Like lipids, synthetic amphiphilic surfactants possess a hydrophilic head and a hydrophobic tail, enabling them to self-assemble into thin monolayers^26^. In fluorosurfactants, the hydrocarbon tail is replaced by a fluorocarbon chain, which significantly increases interfacial tension (26–31 mN/m for fluorosurfactants *vs.* 7–16 mN/m for non-fluorosurfactants)^27^. This higher interfacial tension promotes uniform size distribution and results in smaller PCND diameters. However, a major limitation of PCND formulated with surfactants such as ZONYL-FSO, Span 20, or Pluronic F68 is their reduced stability compared to lipid-based PCND. Surfactant-coated PCND were shown to remain stable in the storage solution for only 30–60 min after synthesis and began to coalesce within 2–3 h, leading to increased PCND size^28^. In contrast, lipid-coated PCND remained stable for approximately 4 h and exhibited only minor size increases thereafter^25^.

Serum albumins are also commonly used as protein-based shell stabilizers for PCND. Human serum albumin (HSA), the most abundant protein in human plasma, has a physiological concentration of 35–50 mg/mL and a blood half-life of up to 19 days^29^. These properties have made HSA a key component in the formulation of early clinically approved microbubble formulations, such as air-filled Albunex® and fluorocarbon-filled successor, Optison®^2^. Similarly, bovine serum albumin (BSA) has been frequently used in PCND formulations^30^. BSA closely resembles HSA, sharing a similar molecular weight (∼66.5 kDa), disulfide bond pattern, and 76% structural identity. Its low cost makes BSA attractive for formulation development and experimental studies of PCND^31^. However, the non-human origin of BSA goes along with immunogenicity risks in clinical applications. Using HSA improves translational safety and regulatory acceptance, as previously demonstrated with Abraxane, a clinically approved cancer nanomedicine, which consists of HSA nanoparticles loaded with paclitaxel^32^.

Polymer-based shells, constructed from materials such as poly(lactic-*co*-glycolic acid) (PLGA), poly(caprolactone) (PCL), poly(lactic acid) (PLA) and polydopamine (PDA), offer enhanced rigidity and superior shelf-life stability at 4 °C compared to lipid-coated PCND^33^. In addition, amphiphilic block copolymers such as polyethylene glycol (PEG)-*b*-PCL, composed of hydrophobic and hydrophilic segments in a defined sequence, promote emulsification of the fluorocarbon core through hydrophobic interactions, while the hydrophilic segments provide steric stabilization in the aqueous phase^34^. However, their increased shell rigidity raises the vaporization threshold, requiring more intense ultrasound exposure for phase transition than their lipid- or protein-coated counterparts^25^^,^^33^.

### 2.3 Synthesis routes

The PCND synthesis toolbox includes various techniques, including homogenization, sonication, microfluidic techniques, condensation, extrusion, and spontaneous nucleation, with the choice of method depending on the specific formulation^69^. Emulsification-based methods—such as sonication and homogenization—are widely used for PCND synthesis due to their simplicity, accessibility of homogenizers, and scalability. Specifically, sonication employs high-frequency sound waves to disperse the fluorocarbon phase into the aqueous medium, while homogenization applies high shear forces to generate more uniformly sized PCND with improved monodispersity. While effective, these methods can lead to broad size distributions if process parameters are not carefully controlled^70^. In contrast, microfluidic synthesis enables precise control over PCND size and monodispersity by manipulating fluid flow within microscale channels^53^^,^^71^^,^^72^. While this method offers high reproducibility, its low throughput limits its applicability to research settings rather than large-scale production^33^. The condensation method first requires the synthesis of microbubbles with fluorocarbon gas cores, which are then condensed into submicron PCND by precisely lowering the ambient temperature and increasing the ambient pressure^45^^,^^73^. However, achieving a narrow size distribution at the nanoscale is challenging, as the process begins with highly polydisperse lipid-coated microbubbles^45^^,^^74^. Extrusion involves forcing an emulsion through a membrane with defined pore sizes, resulting in improved monodispersity compared to sonication and homogenization, which is particularly effective for achieving uniform size distributions^75^. Spontaneous nucleation methods, such as phase inversion and solvent evaporation, rely on physicochemical changes to induce droplet formation. While these approaches can yield highly uniform PCND, they require precise control over solvent removal and concentration gradients, making them more complex than emulsification-based techniques^44^^,^^76^. A comprehensive overview of synthesis techniques can be found in a review by Sheeran et al.^75^.

### 2.4 Functionalization

Surface functionalization of the PCND shell plays a critical role in prolonging circulation time and enabling targeted delivery. This can be achieved through covalent modifications, such as ligand conjugation^77^, or through non-covalent interactions, including hydrophobic^78^^,^^79^, electrostatic^80^, and avidin-biotin binding^77^.

To prolong circulation, PEGylation is commonly applied by introducing PEG chains onto the PCND outer shell, forming a hydrophilic barrier that minimizes opsonization and reduces immune recognition during PCND circulation in the blood pool^81^. In addition, direct coating PCND with red blood cell membranes, which retain key surface proteins such as CD47, is also possible. This “self-marker” protein inhibits phagocytosis by macrophages, thereby extending circulation time^78^^,^^79^.

PCND can also be functionalized with various ligands, including small molecules (*e.g.,* folic acid), peptides, and antibodies, to enable selective binding to disease-specific biomarkers (Table 2). Tumor-targeted strategies primarily fall into three categories: vascular targeting, direct cancer cell targeting, and hypoxic region targeting. PCND designed for vascular targeting focus on markers of tumor angiogenesis. For instance, functionalization with cyclic RGD peptides enables binding to *α*_v_*β*_3_ integrin, a key angiogenic marker overexpressed on endothelial cells^50^^,^^51^^,^^78^^,^^82^.

**Table 2:** Selected active targeting ligands involved in PCND formulations.

Ligands/specific molecule	Specific antigens or receptor	Shell material	Indication	Ref.
Plaque-targeted PP1 peptide	Class A scavenger receptors	Polymer	Macrophages in the rupture-prone plaque	86
Fibrin-targeted peptide	Fibrin	Lipid	Thrombus	88
HA	CD44, the receptor for hyaluronate-mediated motility	Hybrid (lipid and polymer)	Hepatocellular carcinoma	80
Folic acid	Folic acid receptor	Polymer	Nasopharyngeal carcinoma	18
Glycyrrhetinic acid	Glycyrrhetinic acid receptors	Lipid	Human liver cancer tumor model	84
Antibody or Herceptin/anti-HER2/neu peptide	HER2	Polymer	HER2-overexpressing breast cancer	77,83
cRGD	Integrin *α*_v_*β*_3_	Hybrid (lipid and RBCM)	Tumor angiogenesis	50,51,78,82
Bifidobacterium	N/A	Lipid	Hypoxic zone of tumor	62
VCAM -1-targeted peptide	VCAM-1	Lipid	The vascular cells of biofilm-associated surgical site infection	87

Abbreviations: cRGD, cyclic Arg-Gly-Asp peptide; HA, hyaluronic acid; HER2, human epidermal growth factor receptor 2; RBCM, red blood cell membrane; VCAM-1, vascular cell adhesion molecule-1; N/A, not available.

For direct cancer cell targeting, PCND can be conjugated with antibodies or peptides that recognize receptors overexpressed on tumor cells. For example, PCND modified with anti-HER2 antibodies or HER2-binding peptides can recognize and engage with HER2-overexpressing breast cancer cells, potentially enhancing molecular imaging and cargo delivery^77^^,^^83^. Similarly, hyaluronic acid (HA)-modified PCND target CD44 receptors, potentially enhancing the retention of the nanoformulation in the tumor area^80^. Folate-conjugated PCND target folate-receptors-overexpressing cancer cells and facilitate intracellular uptake in nasopharyngeal carcinoma^18^. In addition, glycyrrhetinic acid-modified PCND accumulate strongly in hepatocellular carcinoma cells and improve the cytotoxic efficacy of chemotherapeutic agents^84^. However, despite enhanced receptor engagement and cellular uptake, active targeting often compromises nanoparticle stability and blood circulation time. Ligand modification may accelerate clearance by the mononuclear phagocyte system and reduce tumor accumulation and therapeutic efficacy. Here, a meta-analysis suggests that, in many cases, actively targeted nanoparticles do not (or only marginally) outperform passive targeting in terms of tumor delivery^85^.

In hypoxic regions of solid tumors, the facultative anaerobic *Bifidobacterium* can colonize. Positively charged PCND can attach to the negatively charged surfaces of *Bifidobacterium* and tumor cell membranes *via* electrostatic interactions, enhancing deposition and retention within the tumor microenvironment^62^.

Beyond tumor targeting, several ligand-modified PCND formulations have shown promise in other disease areas. For instance, PCND targeting class A scavenger receptors can accumulate in rupture-prone atherosclerotic plaques, supporting their application in anti-atherosclerosis therapy^86^. Additionally, PCND functionalized with peptides targeting vascular cell adhesion molecule-1 (VCAM-1) can selectively bind to endothelial cells at sites of biofilm-associated surgical site infections. This targeting facilitates transcytosis across tight vascular barriers *via* VCAM-1-mediated pathways, allowing the nanocarriers to penetrate biofilms and enhance therapeutic efficacy^87^.

Altogether, these examples highlight the potential of ligand-modified PCND to improve imaging, drug delivery, and therapeutic efficacy across diverse disease models. The choice of functionalization strategy should be guided by the desired circulation profile and target specificity to ensure optimal performance in biomedical applications.

## 3 *In vivo* fate of phase change nanodroplets

To optimize the therapeutic efficacy of PCND, the biological effects induced by the change of PCND size during vaporization at the target site^89^ need to be comprehensively understood. Furthermore, their circulation time, biodistribution, and clearance mechanisms can determine their effectiveness as contrast agents and drug carriers. Techniques, such as gas chromatography–mass spectrometry (GC–MS), fluorescence imaging (FI), photoacoustic imaging, positron emission tomography (PET), magnetic resonance imaging (MRI), nuclear magnetic resonance spectroscopy, and optical imaging, enable precise tracking of labeled or unlabeled PCND and shed light on their interactions with the mononuclear phagocyte system (MPS) and clearance kinetics.

### 3.1 Blood circulation times

Following intravenous administration, PCND interact with plasma proteins, leading to opsonization and subsequent uptake by MPS cells^90^. Meanwhile, some PCND extravasate into tissues and are internalized by cells^91^. Cellular uptake and tissue extravasation, along with the subsequent clearance, are influenced by core material, shell composition, and particle size (Table 3).

**Table 3:** The half-life of PCND is dependent on their composition.

Core	Shell	Mean diameter	Half-life	Test method	Ref.
C_3_F_8_	Lipid	154 ± 64 nm	3.7 ± 1.1 min	Ultrasound (kidney)	35
C_4_F_10_	Lipid	166 ± 59 nm	10.8 ± 1.6 min	Ultrasound (kidney)	35
C_4_F_10_	Lipid	560 ± 10 nm	19 ± 10 min	GC–MS	45
C_4_F_10_	Lipid	560 ± 10 nm	38 ± 6 min	Ultrasound (vena cava)	45
C_4_F_10_	Lipid	560 ± 10 nm	37 ± 9 min	Fluorescence intensity	45
C_4_F_10_	Lipid	155 ± 4 nm	26.3 ± 16.0 min	Ultrasound (kidney)	38
C_4_F_10_	Lipid	124 ± 4 nm	16.6 ± 9.8 min	Ultrasound (kidney)	38
C_4_F_10_	Lipid	164 ± 9 nm	2.6 ± 0.8 min	Ultrasound (kidney)	38
C_6_F_14_	Surfactant	∼175 nm	12 min	^18^F–PET	64
C_6_F_14_	Lipid	205 ± 2 nm	43.3 min	GC–MS	92
C_10_F_20_O_5_	Protein	160 nm	300 min	GC–MS	68
C_10_F_20_O_5_	Polymer	275 ± 50 nm	120 min	^19^F–MRI	24
C_10_F_20_O_5_	Polymer	–	360 min	^19^F–MRI	24
C_10_F_20_O_5_	Polymer	∼270 nm	240–300 min	Fluorescence intensity	79
C_10_F_20_O_5_	Lipid	∼83 nm	360–600 min	^19^F–MRI	93

*Abbreviations:* GC–MS, gas chromatography–mass spectrometry; PET, positron emission tomography; MRI, magnetic resonance imaging.

*Note:* The standard deviation was not reported in some studies (*e.g.*, 24,64,68,92).

**Figure 2:**
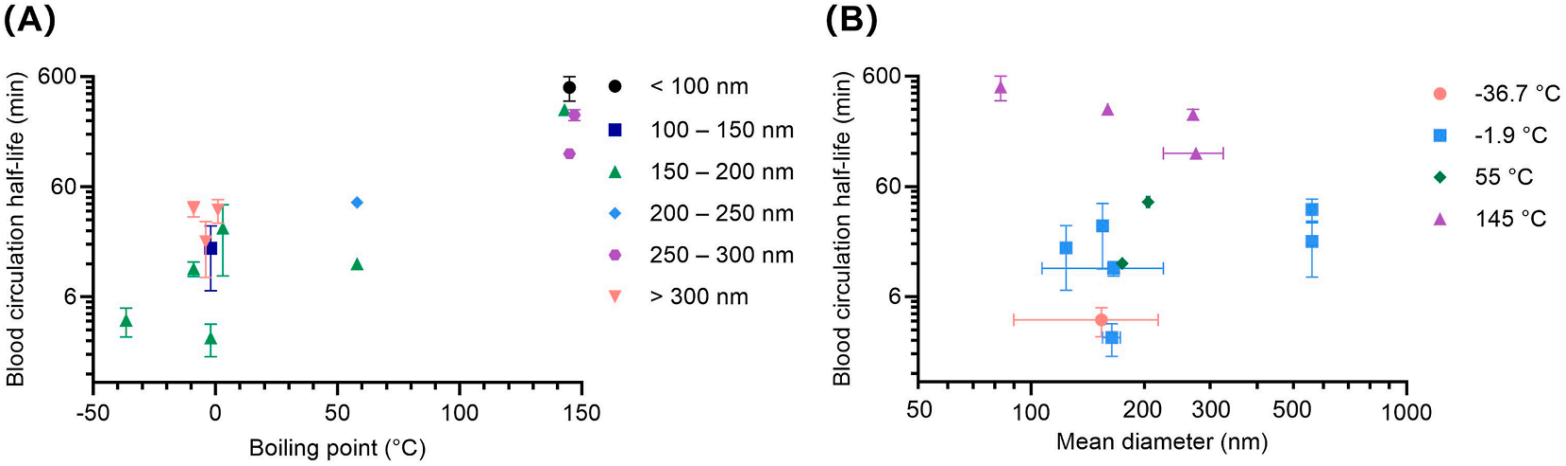
Blood circulation half-life of phase change nanodroplets (PCND) is jointly influenced by particle size and core boiling point. Data points represent literature-reported PCND blood circulation half-lives summarized in Table 3 (*n* = 15); SD values were not available for all entries. (A) PCND half-lives plotted against core boiling point; dot color represents particle size, where points are slightly displaced along the *X*-axis at boiling points of −1.9 °C and 145 °C for visualization purposes only. (B) PCND half-lives plotted against particle size; dot color represents the boiling point of the core material.

To quantitatively assess how physicochemical factors influence PCND circulation, we summarized literature-reported blood circulation half-life data (Table 3). PCND with the lowest boiling point core (C_3_F_8_) shows very short half-lives (3.7 min, *n* = 1)^35^, while C_4_F_10–_based formulations exhibit a broader range (2.6–38 min, *n* = 7, median = 19 min) (Fig. 2A)^35^^,^^38^^,^^45^. Two C_6_F_14_ PCND were reported to have intermediate half-lives (12–43 min; median = 27.7 min)^64^^,^^92^, whereas droplets with high-boiling-point C_10_F_20_O_5_ cores remained in circulation for 120–600 min (*n* = 5, median = 300 min)^24^^,^^68^^,^^79^^,^^93^. Overall, the increase in mean half-life from approximately 4 to 300 min shows that the circulation time is strongly dependent on the core boiling point. Partial overlap exists between C_4_F_10_ and C_6_F_14_ formulations, suggesting that factors beyond the core boiling point, such as shell composition or PCND size, also influence the circulation behavior. Among C_4_F_10–_based PCND, Yoo et al.^38^ reported that the lipid chain length significantly affected the half-life of PCND. Shorter chains (*e*.*g*., DPPC) result in short half-lives (2.6 ± 0.8 min), whereas longer chains (*e*.*g*., DBPC) prolong circulation to 26.3 ± 15.6 min by improving PCND stability and reducing dissolution. In C_10_F_20_O_5_ PCND, smaller particles (>100 nm) had longer half-lives (360–600 min)^93^, while larger particles (>200 nm) were more rapidly cleared from the blood (half-life 120–300 min) by the MPS, as shown in Fig. 2B^24^^,^^79^. In addition to these formulation-dependent factors, differences in analytical methodology contribute to apparent discrepancies. Melich et al.^45^ demonstrated that the detection techniques resulted in varying half-life measurements for the same PCND formulation. Specifically, half-lives measured by fluorescence intensity were longer than those measured by GC–MS. This discrepancy arises because fluorescence detection reflects the persistence of labeled shell components, whereas GC–MS quantifies the core fluorocarbon content.

### 3.2 Organ biodistribution

Liver and spleen are the dominant sites of PCND accumulation due to MPS uptake^11^^,^^34^^,^^45^^,^^87^^,^^94^, ^95^, ^96^, ^97^ (Table 4). Despite this, particle size can strongly affect their biodistribution patterns. For example, PCND in the 100–200 nm size range display lower uptake by macrophages in the liver and spleen, and overall accumulations in these organs, than PCND with sizes below 100 nm^96^. Lung accumulation also varies with size, but in a non-linear manner: very small PCND (∼33 nm) have an extremely low lung accumulation whereas intermediate-sized particles (∼100 nm) have high lung uptake^98^. Larger particles (>200 nm) again have lower lung accumulation^17^^,^^18^^,^^99^. These trends highlight the complex interplay between particle size and organ-specific clearance mechanisms. The accumulation of PCND in the kidneys is moderate compared to these organs, about half as high as in the liver^11^^,^^18^^,^^34^^,^^51^^,^^63^^,^^80^^,^^99^.

**Table 4:** Overview of biodistribution studies reported for PCND.

PCND formulation (core + shell material)	Mean diameter	Imaging label	Distribution	Test method	Ref.
C_4_F_10_ + lipid	560 ± 10 nm	Cy5.5	Mainly in the liver and spleen	FI	45
C_4_F_10_ + polymer	∼800 nm	–	Mainly in the liver and spleen; minor in the kidneys.	Ultrasound	46
C_5_F_12_ + lipid	208 ± 23 nm	Cy5	Mainly in the liver and spleen, kidneys; less in the lungs, intestine and heart (24 h)	FI	87
C_5_F_12_ + lipid	318 ± 149 nm	IR800	Mainly in the liver and spleen, less in the lungs, kidneys and heart (24 h)	FI	34
C_5_F_12_ + lipid	245 ± 10 nm	DiR	Mainly in the liver and spleen; less in the lungs, kidneys and heart (24 h)	FI	80
C_5_F_12_ + polymer	216 ± 63 nm	DiR	Mainly in the liver and spleen; less in the lungs, kidneys and heart (2 h)	FI	18
C_5_F_12_ + polymer	<100 nm	Poly epinephrine	Mainly in the liver and spleen (4 h)	FI	96
C_5_F_12_ + lipid	330 ± 62 nm	Cy5.5	Mainly in the liver, less in the kidneys, spleen, intestine and heart (24 h)	FI	51
C_5_F_12_ + polymer	357 ± 15 nm	DiR	Mainly in the liver and spleen	FI	25
C_5_F_12_ + polymer	409 ± 25 nm	DiR	Mainly in the liver and spleen	FI	25
C_5_F_12_ + lipid	<100 nm	DiR	Mainly in the liver and spleen (6 h and 24 h)	FI	100
C_5_F_12_ + lipid	205 ± 17 nm	ICG	Mainly in the liver, less in the kidneys and lungs (24 h)	FI	99
C_5_F_12_ + lipid	332 ± 14 nm	DiR	Mainly in the liver and spleen (2 h)	FI	97
C_6_F_14_ + surfactant	∼266 nm	FITC	Mainly in the kidneys, less in liver; Both decreased over 24 h; minor in the heart, spleen and lungs	FI	17
C_6_F_14_ + surfactant	∼175 nm	^18^F	Liver accumulation decreased over 2 h; spleen peaked at 45–65 min, then cleared rapidly	^18^F-PET	64
C_6_F_14_ + surfactant	33 nm	Gadolinium	Mainly the in liver and spleen; less in the kidneys, heart and lungs	T_1_-MRI	11
C_6_F_14_ + lipid	106 nm	IR780	Mainly in the liver and lungs (24 h)	FI	98
C_6_F_14_ + lipid	300 nm	DiR	Mainly in the liver and spleen; less in the lungs, kidneys and heart (48 h)	FI	63
C_6_F_14_ + polymer	239 ± 6 nm	Ce6	Mainly in the liver and spleen	FI	101

*Abbreviations:* Ce6, chlorin e6, a fluorescent dye with emission at 668 nm; Cy5, cyanine 5, a fluorescent dye with emission at 670 nm; Cy5.5, cyanine 5.5, a fluorescent dye with emission at 695 nm; DiR, 1,1ʹ-dioctadecyl-3,3,3ʹ,3ʹ-tetramethylindotricarbocyanine iodide, a fluorescent dye with emission at 778 nm; FI, fluorescence imaging; FITC, fluorescein-5-isothiocyanate, a fluorescent dye with emission at 516 nm; IR780, infrared 780, a fluorescent dye with emission at 792 nm; IR800, infrared 800, a fluorescent dye with emission at 792 nm; ICG, indocyanine green, a fluorescent dye with emission at 813 nm; MRI, magnetic resonance imaging; PET, positron emission tomography.

*Note:* The timepoint means the time interval after PCND injection.

### 3.3 Clearance

**Figure 3:**
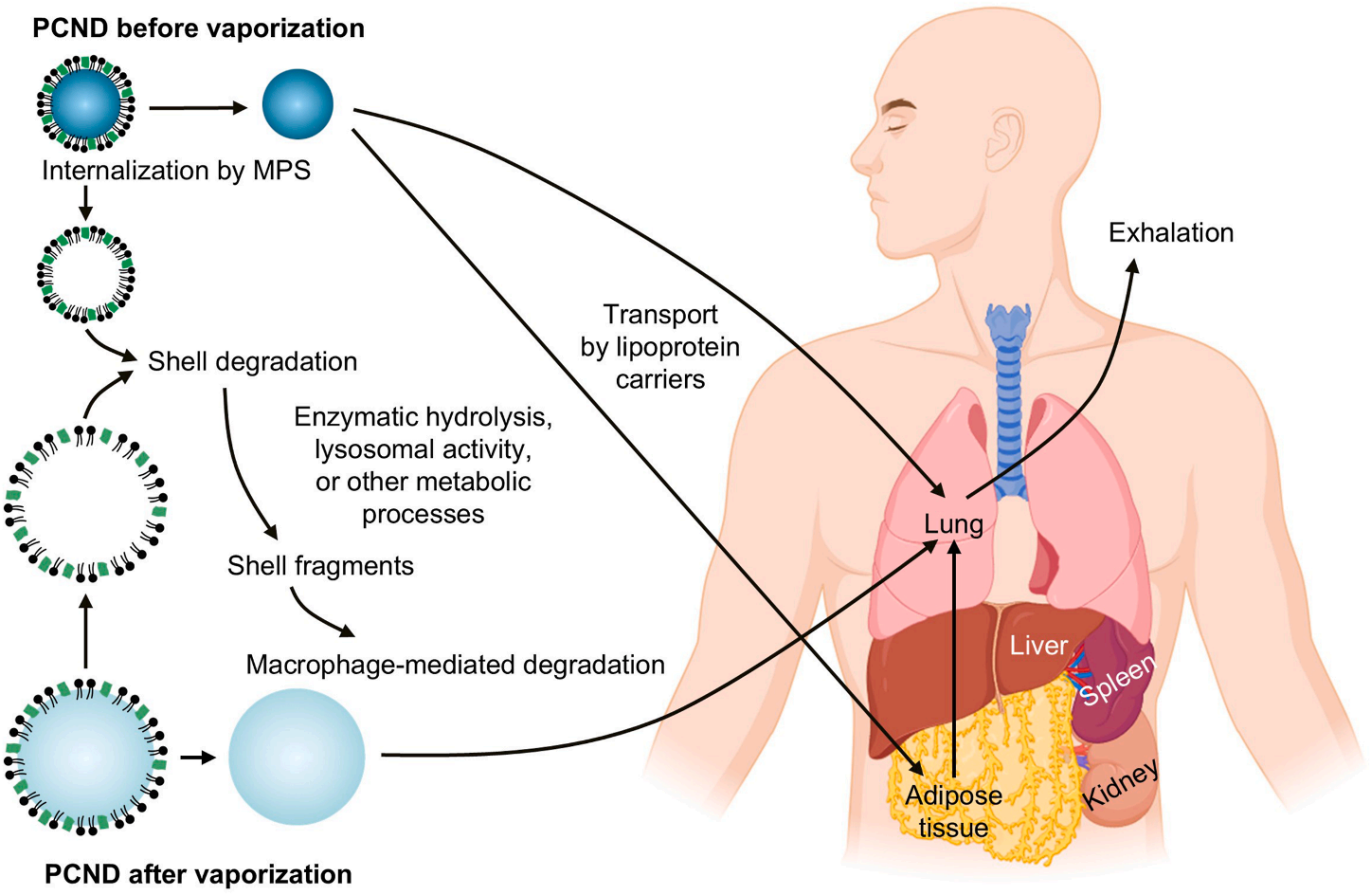
Clearance pathways of Phase change nanodroplets (PCND). Pre- and post-vaporized PCND follow distinct clearance routes. PCND are taken up by the mononuclear phagocyte system (MPS), where the shell undergoes degradation, and the fluorocarbon core is either exhaled or redistributed based on solubility. After vaporization, the gaseous fluorocarbon is rapidly cleared through the lungs, while residual shell components are metabolized in major MPS organs. These routes are influenced by the physicochemical state and composition of the PCND.

Due to the lack of specific studies on the *in vivo* metabolism of PCND, their clearance pathways, which probably differ between the pre- and post-vaporized states, are inferred from fluorocarbon nanoemulsions and microbubbles (Fig. 3), which share similar materials and size-dependent behaviors. Prior to vaporization, the nanometer-sized PCND are internalized by monocytes in the blood or macrophages in the tissue. The degradation of the PCND shell has not been investigated well, but it should be comparable to that of other nanoparticles using the same materials. Therefore, shell degradation may occur through lysosomal enzymatic hydrolysis or other metabolic pathways, followed by the excretion of byproducts, such as monomers or small molecules^102^. For example, polymeric shells are cleaved by enzymes, whereas lipid shells are hydrolyzed by lysosomal acid lipase. Protein shells, such as albumin, are degraded into peptide fragments in lysosomes throughout the body, and kidney-filtered fragments are reabsorbed and hydrolyzed into amino acids before re-entering systemic metabolism^103^. The released fluorocarbon core is cleared *via* the lungs, and the gas is exhaled. How fast fluorocarbon is exhaled depends on the PCND degradation site. After systemic administration, most PCND are internalized by macrophages in MPS organs; fluorocarbon cores are released into the bloodstream by the phagocytic cells and transported by lipoprotein carriers due to their lipophilicity. More hydrophilic fluorocarbons are rapidly cleared *via* the lungs^104^, whereas less soluble fluorocarbons may temporarily accumulate in adipose tissue before slowly redistributing into the circulation for eventual elimination through the lungs^90^^,^^105^^,^^106^. Additionally, the uptake of circulating PCND by blood monocytes has been reported, which subsequently migrate to the pulmonary alveoli and contribute to fluorocarbon clearance. However, this clearance pathway is considered minor compared to uptake by macrophages in MPS organs^105^.

After vaporization, PCND are transformed into micrometer-sized bubbles, changing their clearance dynamics. The vaporized fluorocarbon is then rapidly eliminated through pulmonary exhalation, a much faster process than the MPS-mediated clearance of non-vaporized ones. The clearance rate of fluorocarbon is influenced by their vapor pressure and molecular weight, with lower molecular weight fluorocarbon being excreted more rapidly^90^. The fate of the shell after vaporization is expected to resemble that of non-vaporized PCND (macrophage-mediated degradation and clearance through the liver, spleen, and renal pathways). These complex clearance pathways highlight the influence of PCND states, compositions, and physicochemical properties on their biodistribution and excretion, and represent a difficult task for fulfilling regulatory required kinetic tests for clinical translation.

### 3.4 Safety profiles

Although safety data for PCND are limited (as the field is still at the preclinical stage of development), the use of similar fluorocarbon-based materials allows cautious extrapolation from fluorocarbon nanoemulsion studies. In a Phase Ib/II stroke trial (*n* = 24), a C_5_F_12_ nanoemulsion was administered intravenously at 0.001, 0.002, and 0.0034 g/kg, with no dose-limiting toxicities and no significant differences in adverse events, such as cough, headache, hypertension, and musculoskeletal pain, compared to placebo^107^. Although not ultrasound-activated, the shared fluorocarbon chemistry provides a clinically relevant safety context. In line with this, preclinical toxicity studies of fluorocarbon nanoemulsions indicate a high safety margin, with lethal doses ranging from 25 to 54 g fluorocarbon/kg and no-adverse-effect levels between 2.7 and 9 g fluorocarbon/kg^90^, both well above typical application doses of PCND (lower than 0.2 g/kg^62^^,^^98^). However, unlike nanoemulsions, PCND are vaporization-enabled agents, and their behavior *in vivo* is influenced not only by chemical composition but also by physical phase transitions. In particular, low-boiling-point fluorocarbon cores exhibit higher volatility and are more prone to rapid phase change under physiological conditions, potentially resulting in distinct and more acute biological responses. This means the safety thresholds derived from nanoemulsion studies may not directly apply to PCND, especially those formulated with low-boiling-point components.

One notable vaporization-related phenomenon is increased pulmonary residual volume (IPRV), observed in some animal models following intravenous nanoemulsion injection. IPRV is characterized by impaired lung deflation due to air trapping and is thought to result from fluorocarbon vaporization across the alveolar–capillary barrier and interactions with pulmonary surfactants, leading to foam formation. Its severity appears to correlate with fluorocarbon volatility, with more volatile cores inducing stronger effects. Susceptibility also varies by species—rabbits, pigs, and monkeys are more sensitive (*e*.*g*., 8.1 g fluorocarbon/kg in rabbits, 5.4 g in pigs, and 8.1–10.8 g in monkeys), while dogs, rats, and mice show higher tolerance. Histological analysis often reveals only mild interstitial changes, and IPRV has not been reported in humans, possibly due to anatomical and physiological differences such as larger alveoli, higher transpulmonary pressures, and more effective clearance mechanisms^90^.

In addition to volatility-driven effects, the ADV process itself can also induce biological effects, which are primarily governed by the acoustic parameters that control vaporization dynamics. In rat kidneys, low-boiling-point PCND were well tolerated at moderate mechanical index (MI = 0.8–1.35), whereas activation at the clinical upper limit (MI = 1.9) induced acute tubular hemorrhage and reversible elevations in serum creatinine, with full histologic recovery within 2–4 weeks. Control experiments confirmed that ultrasound exposure alone, even at comparable or higher intensities, caused no renal damage^108^, confirming that tissue effects arise primarily from ADV and cavitation.

Immunogenicity of shell materials is another important safety consideration. BSA, commonly used in preclinical PCND formulations, is subject to regulatory scrutiny due to theoretical bovine spongiform encephalopathy-related risks^109^, and its intrinsic immunogenicity. Although elevated anti-BSA antibodies have been reported without associated clinical symptoms^110^, its non-human origin makes it unsuitable for repeated systemic dosing. In addition, avidin-biotin coupling systems, including the widely used streptavidin-biotin variant for ligand attachment, can be associated with substantial immunogenicity^111^. Even clinically approved excipients such as PEG can induce anti-PEG antibodies in a significant portion of the population, potentially accelerating clearance or causing infusion reactions^112^. These studies outline important aspects of immunogenicity; they leave open several fundamental questions regarding dose thresholds and long-term biological effects.

Regarding organ responses, histological examinations of the liver have shown mild sinusoidal dilatation and macrophage accumulation in granulomas, indicating a sub-clinical inflammatory response potentially related to PCND inflow and hepatic uptake^46^. Overall, animal studies report good *in vivo* tolerability of PCND at appropriate doses, with no evidence of microvascular obstruction, organ toxicity, hemolysis, or discomfort^11^^,^^46^^,^^58^.

Nonetheless, these studies characterize only acute and formulation-specific safety aspects, while leaving critical long-term questions unresolved. Systematic evaluations of maximum tolerated dose, organ-specific toxicity, and the effects of chronic exposure, repeated dosing, and long-term organ accumulation are still lacking, hindering the establishment of reliable safety margins for clinical translation.

### 3.5 Comparison with microbubbles and nanoparticles

Due to their size and phase change characteristics, PCND exhibit nanoparticle-like properties before vaporization and microbubble-like properties thereafter. Since both reference systems already have clinically implemented equivalents, comparative studies with both systems are crucial for elucidating their biological behavior and as a guide for clinical translation of PCND.

#### 3.5.1 Comparison with microbubbles

Commercial microbubbles such as Sonovue® and Definity®, ranging from 1 to 10 μm in size, remain confined to the vasculature with minimal extravascular accumulation^113^, ^114^, ^115^. They remain in the bloodstream for only a few minutes, depending on the gas core composition and shell material: for instance, Sonovue® (sulfur hexafluoride gas, lipid shell) has a half-life of around 6 min^116^, while Definity® (C_3_F_8_ gas core, lipid shell) lasts 79 ± 25 s^117^. Because PCND are nanoscale prior to vaporization, their biodistribution and clearance differ significantly from microbubbles.

#### 3.5.2 Comparison with nanoparticles: nanobubbles, liposomes, protein and polymeric agents

Nanoparticles (1–100(0) nm) are typically opsonized and cleared *via* the MPS, similar to PCND^118^. Nanobubbles (200–500 nm), which possess a gaseous core stabilized by a shell, have been developed for ultrasound imaging and drug delivery beyond the vascular compartment^119^. Nanobubbles typically have a circulation half-life of about 2 h^120^, which may exceed the half-life of PCND with low-boiling-point cores (C_3_F_8_, C_4_F_10_, C_6_F_14_; −36.7 °C to 56 °C) as PCND tend to vaporize, expand, and be cleared rapidly. In contrast, PCND with high-boiling-point cores (C_10_F_20_O_5_; 145 °C) display slower vaporization, therefore remaining in the nanoscale range and achieving prolonged circulation comparable to or longer than that of nanobubbles. Nanoparticles with similar shell materials—such as liposomes^121^, ^122^, ^123^, ^124^, ^125^, polymer-based nanoparticles^126^, and protein-shelled nanoparticles^127^—generally exhibit significantly longer circulation times than PCND. This discrepancy is primarily due to the instability of the liquid fluorocarbon core of PCND, which predisposes them to rapid clearance or vaporization after injection. Thus, low-boiling-point cores limit the stability of PCND *in vivo*, and they also pose challenges for storage stability, indicating that improving robustness without sacrificing acoustic responsiveness should be the focus of further translational research.

### 3.6 Tumor accumulation

Understanding the dynamics of PCND accumulation in tumors is essential for optimizing diagnostic and therapeutic interventions, particularly in determining the optimal time window for imaging and drug release. While most studies reported an increase in tumor accumulation within 1–12 h post-injection, often peaking 24 h or even 48 h post-injection^18^^,^^56^^,^^62^^,^^96^^,^^100^^,^^128^^,^^129^, some studies have observed a different pattern. For instance, Xavierselvan et al.^99^, Wang et al.^17^, and Hu et al.^101^, working with PCND with sizes around 200–300 nm, reported rapid tumor accumulation within the first few hours, followed by a gradual decline over 24 h. Among these patterns, the commonly reported strong tumor concentrations at 24–48 h appear inconsistent with the short systemic half-lives (<45 min) reported for most PCND formulations. One possible explanation for this discrepancy lies in what is being tracked in each type of study. Tumor accumulation studies (Table 5) typically monitor fluorescence signals from labeled shell components, whereas half-life assessments (Table 3) often focus on the clearance of the fluorocarbon core using methods like GC–MS. Since the shell and core may be processed and eliminated through distinct biological pathways, tracking shell components alone may overestimate the retention of intact PCND in tumor tissue, which calls for cautious interpretation. Moreover, quantitative FI inherently faces challenges, including tissue autofluorescence, light attenuation, and depth-dependent signal loss. Studies may also methodologically differ in image acquisition and data processing, such as background subtraction, region-of-interest normalization, or other calibration methods. These methodological differences can substantially affect the fluorescence signal trends and thus the conclusions about PCND tumor accumulation.

**Table 5:** Tumor accumulation of PCND assessed in rodent models.

PCND formulation (core + shell material)	Mean diameter	Imaging label	Tumor accumulation	Method	Ref.
C_5_F_12_ + lipid	205 ± 17 nm	ICG	Peak accumulation at 1.5 h, decreasing to 24 h	FI	99
C_5_F_12_ + lipid	∼159 nm	Epolight 3072 dye	Significant accumulation at 24 h	Photoacoustic imaging	134
C_5_F_12_ + lipid	61 ± 3 nm	DiR	Increase from 2 to 6 h, plateau from 6 to 24 h, decrease from 24 to 192 h	FI	100
C_5_F_12_ + lipid	61 ± 3 nm	N/A	Increase from 0 to 6 h, plateau from 6 to 24 h	Ultrasound	100
C_5_F_12_ + polymer	∼180 nm	Poly-epinephrine	Constant accumulation from 0 to 4 h	Ultrasound	96
C_5_F_12_ + polymer	∼180 nm	Poly-epinephrine	Strong accumulation from 0 to 12 h, little further accumulation from 12 to 24 h, maximum accumulation at 24 h	FI	96
C_5_F_12_ + polymer	195 ± 21 nm	Cy7	Significant accumulation at 8 h; gradual increase from 8 to 12 h, plateau from 12 to 24 h	FI	56
C_5_F_12_ + polymer	216 ± 33 nm	DiR	Rapid accumulation from 0 to 1 h; gradual increase from 1 to 6 h; plateau from 6 to 24 h	FI	18
C_6_F_14_ + lipid	106 nm	IR780	Prominent tumor accumulation at 24 h	FI	98
C_6_F_14_ + lipid	200 nm	IR780	Maximum accumulation at 24 h	Ultrasound + FI	128
C_6_F_14_ + lipid	280 ± 60 nm	DiR	Maximum accumulation at 48 h	FI	62
C_6_F_14_ + lipid	209 ± 7 nm	Polypyrrole	Maximum accumulation at 24 h	Photoacoustic imaging	129
C_6_F_14_ + lipid	230 ± 4 nm	IR780	Maximum accumulation at 24 h	FI	135
C_6_F_14_ + surfactant	266 nm	FITC	Maximum accumulation at 2 h, then decrease	FI	17
C_6_F_14_ + surfactant	33 nm	Gadolinium	Constant increase from 0 to 6 h, decrease at 24 h	T_1_-weighted MRI	11
C_6_F_14_ + polymer	239 ± 6 nm	Ce6	Rapid increase from 0 to 4 h, gradual decrease from 4 to 24 h	Photoacoustic imaging	101
C_6_F_14_ + lipid	∼300 nm	DiR	Increase from 0 to 2 h, plateau from 2 to 48 h	FI	63
C_6_F_14_ + RBCM	∼529 nm	DiO	Increased within 24 h	FI	78

*Abbreviations:* Ce6, chlorin e6, a fluorescent dye with emission at 668 nm; Cy7, cyanine 7, a fluorescent dye with emission at 779 nm; DiO, dialkylcarbocyanine, a fluorescent dye with emission at 501 nm; DiR, 1,1′-dioctadecyl-,3,3′,3′-tetramethylindotricarbocyanine iodide, a fluorescent dye with emission at 778 nm; FI, fluorescence imaging; FITC, fluorescein-5-isothiocyanate, a fluorescent dye with emission at 516 nm; ICG, indocyanine green, a fluorescent dye with emission at 813 nm; IR780, infrared 780, a fluorescent dye with emission at 792 nm; MRI, magnetic resonance imaging; RBCM, red blood cell membrane; NA, not available.

*Note:* The timepoint means the time interval after PCND injection.

PCND accumulate in solid tumors *via* the EPR and ATR effects^79^, as other nanoparticles do. The EPR effect allows drug carriers to accumulate in the tumor microenvironment (TME) due to the abnormal architecture and increased permeability of the tumor vasculature, as well as the disturbed lymphatic and venous drainage^130^, ^131^, ^132^. Additionally, nanocarriers under 200 nm can pass through the leaky tumor vasculature *via* transvascular transport, a process contributing to ATR in tumors. Larger nanoparticles face restricted transvascular transport^130^, and microbubbles cannot diffuse through leaky tumor vasculature^113^, ^114^, ^115^. Using real-time imaging, Rapoport et al.^91^ showed that PCND extravasated into the tumor tissue, which was characterized by a gradual loss of fluorescence within tumor blood vessels and a simultaneous accumulation of fluorescence in the tumor parenchyma. At the microscopic level, studies have provided insights into the spatial distribution of PCND in tumors over time. Cao et al.^133^ reported that C_5_F_12_ + lipid PCND (mean size 234 nm) gradually extravasated, showing minimal accumulation immediately after injection, with increased localization mainly inside and around blood vessels after 12 h—consistent with the observations by Wang et al.^87^ that PCND remained mainly perivascular at this time point—progressing to extravascular diffusion after 24 h. Extravasation of PCND *via* ATR can be assumed but has not been reported so far.

PCND exhibit a non-uniform distribution within tumor tissue^91^^,^^136^. To overcome this, low-intensity focused ultrasound (LIFU) is employed to enhance both vascular and interstitial permeability through sonopermeation^18^, thereby promoting EPR–mediated accumulation and enabling a more uniform distribution within tumors^91^. Furthermore, the cavitation effect caused by LIFU untightens the tumor stroma and drives PCND deeper into the tissue^51^.

## 4 Biomedical applications

### 4.1 Diagnostic studies

**Figure 4:**
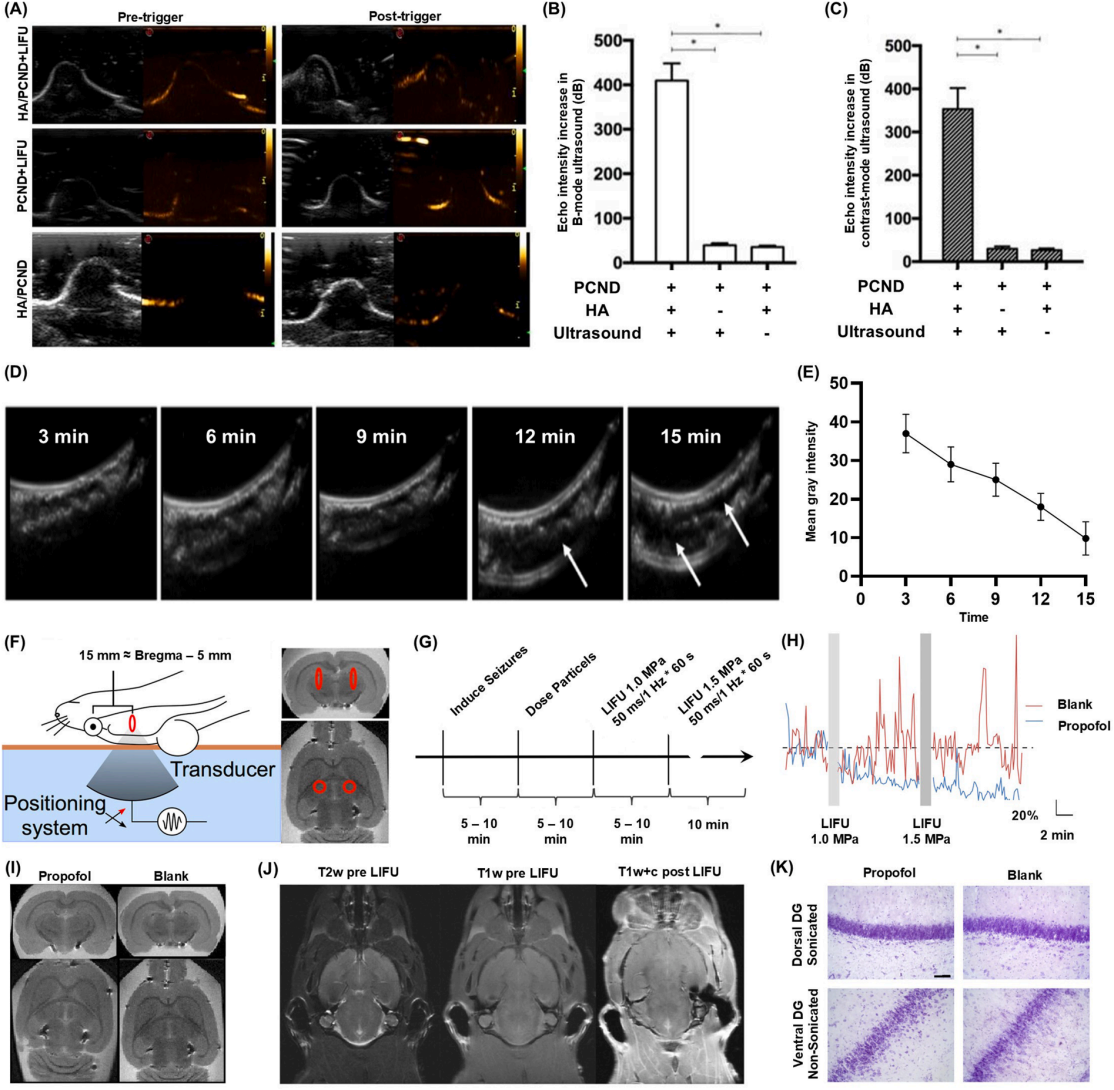
Biomedical applications of phase change nanodroplets (PCND). Panels A–E illustrate diagnostic applications, while panels F–K represent therapeutic application. (A) Representative B-mode (left) and contrast-mode ultrasound images of tumors in mice treated with hyaluronic acid (HA)/PCND + low-intensity focused ultrasound (LIFU), PCND + LIFU, or HA/PCND without LIFU. Prior to administration, all tumors exhibit low baseline echogenicity. One-hour post-injection, LIFU is applied to the HA/PCND + LIFU and PCND + LIFU groups. Only the HA/PCND + LIFU group shows marked post-trigger signal enhancement, indicating efficient accumulation and LIFU-triggered acoustic droplet vaporization. (B, C) Quantitative analysis of echo intensity in B-mode and contrast-mode. Results indicate that HA/PCND accumulated effectively in tumor tissue and underwent acoustic droplet vaporization upon LIFU activation, enhancing imaging signals. These findings demonstrate the potential of PCND for targeted molecular ultrasound imaging. Reproduced from Ref. 80, under CC BY-NC 4.0. Time-dependent thrombolytic efficacy of streptokinase-loaded PCND combined with ultrasound (7.5 MHz probe, mechanical index = 1.2) during sonothrombolysis. (D) Representative ultrasound images of sonothrombolysis progression. (E) Mean grayscale intensity reduction in the region of interest demonstrates enhanced clot dissolution with streptokinase-loaded PCND over 15 min. Reproduced from Ref. 65, under CC BY-NC 3.0. LIFU-triggered propofol release from intravascular PCND suppresses seizure activity *in vivo*, without direct brain penetration or nonspecific tissue effects. (F) The setup consists of a transducer integrated with a stereotactic positioning frame for precise targeting. Those red ellipses represent sonication focuses. (G) Propofol-loaded or blank PCND are administered intravenously, followed by LIFU (2 min, 20% duty cycle) at the seizure focus and real-time neural activity monitoring. (H) Seizure suppression is observed only in rats receiving propofol-loaded PCND + LIFU. *Ex vivo*/*in vivo* magnetic resonance imaging and histology (I–K) reveal no parenchymal damage, confirming the safety of this technique and distinguishing it from other ultrasound-mediated neuromodulation methods that rely on nonspecific brain tissue damage. Reproduced from Ref. 34. Copyright © 2017 American Chemical Society.

PCND enable efficient and targeted tumor imaging through vaporization-triggered contrast, supporting vascular mapping, molecular imaging, and therapy monitoring. After PCND (C_4_F_10_ + lipid) vaporization by focused ultrasound, the generated bubbles can be used to detect tumor-feeding arteries for subsequent tumor embolization^44^. PCND can also be engineered for molecular ultrasound imaging of tumors. For instance, HA- or FA-modified PCND (Fig. 4A–C) enabled vaporization and imaging at target-rich tumor sites, achieving signal enhancement 1 h after injection^11^^,^^80^. For monitoring proton beam radiotherapy, C_4_F_10_ + polymer PCND vaporization by the proton beam enabled the estimation of the proton range *in vivo*^46^.

Beyond tumor imaging, PCND have shown diagnostic value in inflammatory diseases. Ramirez et al.^43^ used contrast-enhanced ultrasound to visualize PCND accumulation in pancreatic islets, correlating with immune infiltration and enabling early detection of presymptomatic type 1 diabetes. Furthermore, Riaz et al.^58^ demonstrated successful cardiac chamber opacification in the ventricles of both rats and dogs, supporting the use of C_5_F_12_ + polymer PCND in echocardiography. C_4_F_10_ + lipid PCND have also been used to image the microvasculature and microcirculation in the spinal cord^76^. However, in this context, Li et al.^76^ reported that conventional B-mode imaging failed to visualize the contrast of the PCND, and assessing PCND specific nonlinear response profiles and suppressing the background using harmonic imaging sequences became essential.

PCND have also been used for ultrasound localization microscopy. *In vitro*, C_5_F_12_ + lipid PCND enhanced the contrast in low-flow microvessels by generating intermittent, high-intensity signals upon vaporization. However, their transient signal limits sustained flow tracking^47^. To address this limitation, PCND were activated by short acoustic pulses and imaged at high frame rates to reconstruct vascular tracks. Using refined acquisition modes—including multi-angle plane-wave compounding and selective activation—*in vivo* studies in rabbit kidneys achieved significant improvements in spatial resolution (resolving vessels at 115 μm spacing) and reduced measurement times to sub-second intervals, enabling phase-specific and branch-selective vascular imaging^137^.

PCND also promote multimodal imaging. For instance, loading PCND with photoabsorbers like near-infrared dyes or gold nanoparticles enables photoacoustic imaging, allowing real-time tracking of PCND and monitoring of vaporization events^10^. This approach also supported lymph node imaging, as PCND can reach lymphatic tissue after intradermal or intravenous injection and provide sustained ultrasound and photoacoustic contrast for up to 72 h^52^. Additionally, by loading PCND with Fe_3_O_4_^138^ or Gadovist^11^, they can function as T_2_-or T_1_-weighted MRI contrast agents, respectively. Besides, fluorocarbons themselves contain ^19^F nuclei, which can be detected by ^19^F MRI with high specificity. This property makes them promising candidates for non-invasive cell tracking, but low-molecular-weight ^19^F tracers in PCND face challenges *in vivo* due to their rapid efflux^139^. Therefore, higher-molecular-weight fluorocarbon, such as C_10_F_20_O_5_, are preferred.

Collectively, these findings demonstrate the broad diagnostic applicability of PCND for assessing vasculature and EPR-related processes. However, it has also become clear that the success of diagnostic PCND approaches depends not only on their contrast-generating capacity but also on their *in vivo* kinetics. Furthermore, the authors believe that for molecular imaging of extravascular targets (*e.g.*, at tumor cells), as shown for liposomes, micelles, and other nanoparticles^140^, the non-specific, EPR-based accumulation may generate a strong background uptake that will make the correct assessment of target receptor expression very difficult.

### 4.2 Therapy studies

#### 4.2.1 Vascular lesions

PCND have shown promise in vascular treatments involving cavitation-induced mechanical sonothrombolysis. Their submicron size allows for deep clot penetration, resulting in faster and more efficient lysis compared to surface-restricted microbubbles^54^. Kim et al.^39^ demonstrated that PCND achieved approximately 140% higher thrombolysis efficiency than compositionally identical microbubbles under ultrasound in an aged bovine blood clot flow model. The enhanced efficacy was attributed to their predominantly intra-clot cavitation compared with microbubbles (internal-to-external clot ratio = 0.38 *vs*. 0.04). Extending this concept, streptokinase-loaded PCND were developed to enhance sonothrombolysis for treating deep vein thrombosis. *In vitro* studies showed a significant reduction in clot weight and hemoglobin content compared with controls (Fig. 4D and E)^65^. Advances in device integration have improved the clinical applicability of PCND. Goel et al.^40^ integrated PCND with a sub-megahertz forward-viewing intravascular ultrasound catheter, achieving localized sonothrombolysis of retracted clots under clinically safe acoustic conditions (0.9 MPa, MI ≤ 1.9). PCND-mediated treatment achieved markedly higher lysis efficiency than all control groups. Clot mass was reduced by ∼30% for PCND + ultrasound and ∼40% for PCND + thrombolytic agent + ultrasound, compared with only 9%–17% for microbubble- or thrombolytic agent-only treatments, underscoring its superior thrombolytic efficacy. In addition to improved efficacy, PCND-mediated thrombolysis also offers enhanced procedural safety. Guo et al.^66^ reported that introducing PCND into ultrasound-mediated thrombolysis resulted in smaller overall clot debris and a markedly reduced volume fraction of large fragments (>10 μm) than microbubbles, minimizing the risk of distal embolization. This effect was attributed to the ability of PCND to penetrate fibrin pores and vaporize locally within the thrombus, enabling fine disruption.

*In vivo* studies have demonstrated the broad therapeutic potential of PCND in thrombolysis. In porcine models, ultrasound-induced PCND cavitation effectively dissolved coronary microthrombi^141^ and deep venous thrombi^37^, yielding over fourfold improvement in clot resolution compared with thrombolytic therapy alone^37^. A strategy of endowing PCND with fibrin-targeting further enhanced clot affinity and ultrasound-mediated reperfusion in a rat hindlimb microvascular obstruction model^88^. Converging evidence from plaque studies, Gao et al.^86^ observed macrophage apoptosis and dispersal of activated platelets within atherosclerotic plaques during PCND cavitation. This suggests a multifaceted therapeutic mechanism involving both physical and biological modulation. Collectively, these findings underscore that PCND are capable of achieving safe, efficient, and targeted thrombolysis across multiple vascular contexts.

#### 4.2.2 Tumor therapy

When activated by high-intensity focused ultrasound (HIFU), PCND can induce tissue ablation through both thermal and mechanical mechanisms. For example, in an *in vitro* study in tissue-mimicking phantoms, selective vaporization of PCND at the acoustic focus improved energy deposition and heating efficiency, producing larger focal lesions (53 *vs*. 1 mm^3^ in controls) while minimizing near-field overheating. In contrast, microbubbles caused substantial off-target surface heating, dissipating most energy away from the focus due to their interaction with the HIFU beam along the entire acoustic path. This focal confinement enabled faster, deeper, and more controlled ablation, potentially reducing treatment time by one-third while improving safety^22^. Furthermore, integration with multi-focus HIFU further amplified focal heating while keeping the pre-focal temperature rise below 6 °C, nearly doubling ablation volume and efficiency^142^.

PCND substantially reduce the pressure threshold required to generate histotripsy. Under short-pulse insonation, Glickstein et al.^143^ demonstrated that effective tissue fractionation could be achieved at pressures as low as 4.1 MPa (*vs*. ∼20 MPa conventionally), entirely within safe acoustic limits (MI < 1.9) in an *ex vivo* liver model. The presence of PCND amplifies local cavitation activity and mechanical stress, leading to efficient tissue disintegration within tumor sites^62^^,^^22^^,^^142^.

Beyond ultrasound, optically driven vaporization of Herceptin-targeted PCND using laser stimulation has been shown to disrupt cancer cell membranes and induce targeted cell death, offering an alternative strategy for image-guided, receptor-specific therapy^83^.

Acoustically activated PCND also modulate immunological responses. For example, Cao et al.^133^ found that at low intensities (0.5–1.0 W/cm^2^) the subtle tissue damage induced local immune cell infiltration. Higher intensities (1.5 W/cm^2^) led to rapid bubble collapse, causing extensive necrosis and stronger immune responses at the lesion margins. In addition, Li et al.^50^ found that LIFU-triggered PCND induced immunogenic cell death by promoting the release of necrosis-associated damage-associated molecular patterns release and calreticulin exposure. These effects enhanced dendritic cell maturation and downstream antitumor immunity. These studies suggest that PCND-mediated ablation can be used to enhance anti-tumor immunity.

In addition to direct ablation and immune modulation, PCND can inhibit tumor growth by selectively disrupting the tumor vasculature. Ultrasound activated antifibrinolytic drug-loaded PCND mediate a cavitation-induced endothelial damage and initiate the extrinsic coagulation cascade, leading to the selective thrombotic infarction of tumors, effectively starving rapidly proliferating tumor tissues^78^.

Furthermore, PCND can act as oxygen reservoirs due to the high solubility of oxygen in liquid fluorocarbon and help to alleviate tumor hypoxia, a major obstacle to many therapies. Accordingly, oxygen-carrying PCND have been shown to enhance multiple oxygen-dependent modalities, including radiotherapy^79^^,^^98^, photodynamic therapy^99^^,^^101^^,^^128^^,^^144^, photothermal therapy^67^^,^^96^^,^^129^, chemo-dynamic therapy^56^^,^^96^, and sonodynamic therapy^42^^,^^50^^,^^135^. These enhancements are generally attributed to increased generation and amplification of reactive oxygen species generation (*e.g.* ^1^O_2_ and OH)^42^^,^^50^^,^^128^^,^^135^, greater radiation-induced DNA damage^79^^,^^98^, and elevated oxidative stress and redox activity^56^^,^^96^ that collectively promote tumor cell death.

Drug delivery is another application field for PCND. Drugs (*e.g.*, doxorubicin or quercetin) loaded in PCND have shown higher serum concentrations, prolonged circulation half-lives, reduced clearance, and greater tumor accumulation (over 3-fold increases 5 h post i.v.) than in their free form, primarily due to the EPR effect and reduced off-target clearance^25^^,^^145^. Moreover, encapsulating drugs like doxorubicin in PCND can improve hemocompatibility, likely by reducing myelosuppression and hemolysis of red blood cells^60^. In addition to improved pharmacokinetics and accumulation, ultrasound plays a pivotal role in optimizing the intratumoral distribution and therapeutic efficacy of PCND-delivered drugs. PCND + LIFU enhanced vascular permeability and promoted deeper and more uniform drug distribution within the TME^146^. It also enhanced drug penetration into poorly perfused tumor cores^49^. In another publication, it was shown that the extravasation distance of doxorubicin from the vessel wall increased from 12 μm to over 100 μm upon ultrasound exposure^146^. Correspondingly, LIFU significantly elevated intratumoral doxorubicin concentrations, confirming its role in facilitating local drug uptake and triggering PCND release^60^. The concept was also successful when PCND were stimulated by a laser^147^. Furthermore, ultrasound-induced PCND cavitation promoted transient permeabilization of cellular and nuclear membranes, facilitating intracellular and intranuclear drug delivery^61^. Besides improving therapeutic efficacy, PCND ultrasound minimized off-target toxicity in cardiac, hepatic, and renal tissues^58^^,^^97^.

Building on their potential for drug delivery, PCND have been engineered to encapsulate chemotherapeutic agents like paclitaxel^24^^,^^59^^,^^136^, docetaxel^11^^,^^25^^,^^49^^,^^57^^,^^60^^,^^129^^,^^148^, or 10-hydroxycamptothecin^80^^,^^138^. These formulations effectively reduced tumor volume, suppressed proliferation, and promoted apoptosis. In a triple-negative breast cancer model, paclitaxel- and doxorubicin-loaded PCND improved drug delivery efficiency and delayed tumor progression^97^. Additionally, hydroxychloroquine-loaded PCND, an autophagy inhibitor, suppressed tumor growth by modulating the autophagic process in tumor cells^17^. In addition, combination with anti-PD-L1 antibodies has expanded PCND’s utility in immunotherapy, improving immune activation and tumor suppression^50^^,^^100^^,^^135^. PCND have also been explored for nucleic acid delivery, including plasmid DNA for gene transfection^77^ and microRNA-122, a key regulator of fatty-acid metabolism whose downregulation contributes to the development of hepatocellular carcinoma^149^.

#### 4.2.3 Disorders of central nervous system

In central nervous system applications, PCND combined with LIFU can induce localized neuromodulation, likely mediated by cavitation-generated mechanical forces transmitted from cerebral vasculature to surrounding brain tissue, activating mechanosensitive pathways^150^. They also confine the action of BBB-permeable drugs to millimeter-scale brain regions. For example, LIFU-triggered release of propofol from C_5_F_12–_cored PCND occurred only within the focal vascular bed, enabling focal drug entry during first-pass perfusion and resulting in seizure suppression without inducing off-target anesthesia (Fig. 4F–K)^34^. PCND can also be operated in regimes where BBB opening is desired, with the magnitude and spatial extent tuned by the core composition. Owing to the low vaporization threshold of C_3_F_8–_cored PCND (MI = 0.24), ADV can be achieved at modest pressures (MI = 0.3–0.5) that are compatible with prolonged and large-volume sonication, enabling broad BBB opening. In contrast, C_4_F_10_-cored PCND require higher vaporization pressures (MI = 1.2), exceeding the pressure tolerated by brain tissue for prolonged sonication (MI ≤ 0.7–0.9). Consequently, high-pressure exposure (MI = 1.2) must be restricted to brief, focal bursts to trigger ADV, followed by lower-pressure pulses (MI = 0.5) to sustain locally generated bubble oscillations, resulting in a highly localized BBB opening^36^.

#### 4.2.4 Biofilm

Bacterial biofilms are responsible for many chronic and relapsing infections. They respond poorly to antibiotics because they contain drug-tolerant persister cells (subpopulations of bacteria that enter a dormant state, surviving antibiotic treatments, even if they are not genetically resistant) and a biofilm matrix that serves as a physical barrier to antimicrobial penetration^151^. To address this, Durham et al.^152^ used PCND combined with ultrasound to disrupt methicillin-resistant *Staphylococcus aureus* biofilms and improve drug penetration, thereby enhancing the effectiveness of antibiotics against persister cells. Choi et al.^153^. further demonstrated that PCND-mediated delivery increased antibiotics accumulation in bacterial membranes and cytoplasm by more than tenfold, reducing the concentration needed for complete persister clearance. This strategy has been adapted to clinically relevant biofilm models. A VCAM-1-targeted PCND was developed for biofilm-associated surgical site infections, where VCAM-1-mediated transcytosis enables PCND to cross the inflamed endothelium and extravasate into infected tissue surrounding the biofilm. Upon ultrasound activation, cavitation, generated by PCND, produces strong mechanical forces that propagate into the adjacent biofilm. These forces disrupt the biofilm extracellular polymeric substance, a microbially secreted extracellular macromolecular matrix that forms the structural scaffold of the biofilm, thereby enhancing drug penetration^87^. Similarly, Dang et al.^48^ developed PCND loaded with chlorhexidine or antibiotics to disrupt mature *Enterococcus faecalis* biofilms in a human tooth model, offering a potential strategy for root canal disinfection. Together, these findings highlight the strong translational potential of PCND-assisted sonotherapy for recalcitrant biofilm infections, although its efficacy and safety in complex *in vivo* environments remain to be fully validated.

### 4.3 Clinical translation status

Although no registered clinical trials currently investigate PCND in patients, several early translational efforts indicate that PCND-based systems are progressing toward clinical evaluation. Examples include pre-clinical programs for *Microvascular Therapeutics* for brain tumor ablation (MVT-101) and ultrasound-mediated thrombolysis (MVT-201)^154^^,^^155^.

In parallel, fluorocarbon nanoemulsions with similar submicron dimensions and fluorocarbon cores as PCND have entered early phase human studies. NuvoX *Therapeutics* is currently conducting three clinical programs using its oxygen-carrying C_5_F_12_ nanoemulsion (NVX-108 for glioblastoma^156^, a Phase Ib/II trial in acute ischemic stroke^107^, and the PROVEN Phase IIb trial for large-vessel-occlusion stroke^157^). While these nanoemulsions are not activated by ultrasound and therefore do not constitute PCND in the strict sense, their reproducible manufacturing, intravenous safety, and regulatory progress provide encouraging precedents for the potential clinical translation of PCND.

## 5 Conclusions

PCND represent a highly versatile platform for ultrasound-mediated diagnostics and therapy. Their nanoscale dimensions, biocompatibility, and triggerable vaporization offer a suite of capabilities ranging from image-guided drug delivery and neuromodulation to oxygenation modulation and transient disruption of biological barriers. Despite encouraging preclinical progress, three key challenges continue to hinder the clinical translation of PCND: (1) inconsistent data on pharmacokinetics, (2) confliction and incomplete safety evaluations, and (3) limited vaporization control under physiological conditions. These challenges largely stem from formulation-dependent properties, for example the complex interplay between core boiling point, shell composition, and droplet size, which together affect both pharmacokinetics and vaporization behavior. As a result, reported circulation half-lives and tumor accumulation profiles diverge across studies, a variability further amplified by different imaging protocols and data analysis methods. Addressing these challenges will require both rational formulation strategies to improve systemic stability and on-demand responsiveness, and the development of standardized pharmacokinetic assessment methods to ensure cross-study comparability. Specifically, actionable steps include establishing application-specific formulation guidelines that account for fluorocarbon core and shell properties as well as adopting fixed pharmacokinetic sampling time points across studies to reduce methodological variability in imaging and data analysis.

Safety remains another critical knowledge gap. In particular, low-boiling-point formulations show greater sensitivity to ambient conditions and may induce distinct biological responses, such as pulmonary effects, not observed with more stable nanoemulsions. This underscores that existing safety thresholds derived from studies on nanoemulsions may not directly apply to volatile PCND formulations. Although preclinical data suggest a generally good safety profile at adjusted doses, systematic evaluation of dose limits, organ-specific toxicity, and long-term safety are still lacking, especially for low-boiling-point cores. This gap must be addressed to ensure safe clinical translation.

Beyond biological and safety considerations, formulation-driven characteristics also present practical manufacturing challenges. The need for precise control of droplet size distribution, encapsulated fluorocarbon content, and shell poses challenges for upscaling, while the metastable nature of superheated cores raises concerns regarding shelf life and handling. Ensuring batch-to-batch reproducibility and establishing GMP-compatible production methods will therefore be essential. In parallel, robust and reproducible ultrasound protocols, tuned to the activation thresholds and vaporization dynamics of each formulation, are required to achieve consistent therapeutic outcomes.

Looking forward, PCND systems have the potential to drive a new generation of precision ultrasound theranostics. Their unique phase-change properties and multifunctionality set them apart from other nanomaterials. Beyond oncology, their applications may extend to targeted neurostimulation, immune modulation, and treatment of vascular or inflammatory diseases. However, translating these promising PCND into clinical practice will require overcoming significant technical and translational challenges through thoughtful design, rigorous evaluation, and coordinated interdisciplinary efforts.

## Author contributions

Xiaoyu Wang: data curation, writing–original draft, writing–review & editing, conceptualization. Roman Barmin: writing–original draft, writi–review & editing, conceptualization; Roger Molto Pallares: writing–original draft, writing–review & editing, conceptualization. Anne Rix: writing–original draft, writing–review & editing, conceptualization. Fabian Kiessling: writing–original draft, writing–review & editing, conceptualization, supervision, funding acquisition.

## Declaration of generative AI in scientific writing

The authors declare that no generative artificial intelligence (AI) or AI-assisted technologies were used in the writing, editing, or preparation of this manuscript.

## Conflicts of interest

The authors declare no conflicts of interest.
